# Physical Effects of Buckwheat Extract on Biological Membrane In Vitro and Its Protective Properties

**DOI:** 10.1007/s00232-015-9857-y

**Published:** 2015-11-18

**Authors:** Aleksandra Włoch, Paulina Strugała, Hanna Pruchnik, Romuald Żyłka, Jan Oszmiański, Halina Kleszczyńska

**Affiliations:** Department of Physics and Biophysics, Wrocław University of Environmental and Life Sciences, Norwida 25, 50-375 Wrocław, Poland; Department of Fruit, Vegetable and Cereal Technology, Wrocław University of Environmental and Life Sciences, Chełmońskiego 37/41, 51-630 Wrocław, Poland

**Keywords:** Buckwheat extracts, UPLC analysis, Red blood cell membrane, FTIR spectroscopy, Dichroism, Biological activity

## Abstract

Buckwheat is a valuable source of many biologically active compounds and nutrients. It has properties that reduce blood cholesterol levels, and so reduces the risk of atherosclerosis, seals the capillaries, and lowers blood pressure. The aim of the study was to determine quantitative and qualitative characteristics of polyphenols contained in extracts from buckwheat husks and stalks, the biological activity of the extracts, and biophysical effects of their interaction with the erythrocyte membrane, treated as a model of the cell. An analysis of the extract’s composition has shown that buckwheat husk and stalk extracts are a rich source of polyphenolic compounds, the stalk extracts showing more compounds than the husk extract. The study allowed to determine the location which incorporated polyphenols occupy in the erythrocyte membrane and changes in the membrane properties caused by them. It was found that the extracts do not induce hemolysis of red blood cells, causing an increase in osmotic resistance of erythrocytes. They affect mainly the hydrophilic region by changing the degree of order of the polar heads of lipids, but do little to change the fluidity of the membrane and its hydration. The results showed also that polyphenolic substances included in the extracts well protect the membranes of red blood cells against oxidation and exhibit anti-inflammatory effect.

**Electronic supplementary material:**

The online version of this article (doi:10.1007/s00232-015-9857-y) contains supplementary material, which is available to authorized users.

## Introduction

The increasing demand for nutraceutic products and gluten-free cereal-based products creates the need for entry into the man’s daily diet of new products. One potential pseudo-cereal with these properties is buckwheat (Li and Zhang 2001). It is a plant known and cultivated for thousands of years in North and East Asia. Currently, buckwheat is grown also in Europe. Its most popular genus is *Fagopyrum esculentum* Moench. This plant is rich in starch and protein (Dizlek et al. 2009). From the mentioned regions, a lot of functional foods containing buckwheat have entered the world market, such as e.g. pasta.

Scientific studies show that buckwheat has antioxidant, antiatherogenic, neuroprotective, and photoprotective effects, as well as cytotoxicity and inhibitory activity against angiotensin-I-converting enzyme and α-amylase (Aoyagi 2006; Hinneburg et al. 2006; Kim et al. 2007; Liu et al. 2008; Pu et al. 2004; Sun and Ho 2005; Wojcicki et al. 1995). It was also shown that the compounds identified in buckwheat seeds lower cholesterol and act favorably on the digestive system (Wronkowska and Soral-Śmietana 2008). It is, therefore, a plant that has a number of beneficial properties for humans, but it is not enough popular and too rarely present in the daily human diet.

The subject of our research is the extracts obtained from husks (BH) and stalks (BS) of buckwheat (*Fagopyrum esculentum* Moench). In the literature, there are no reports regarding the impact of these extracts on biological objects, such as cell or cell membrane. In our earlier publications (Pruchnik et al. 2015), we looked at the impact of BS and BH polyphenolic extracts on fluidity of a one-component model membrane, in particular on the main phase transition temperature of dimyristoylphosphatidylcholine (DMPC). In this work, we present the results of further studies on the extracts of buckwheat. We are continuing research on the impact of the above-mentioned extracts on the physical parameters of the already much more complex biological membrane model, i.e., the red blood cell membrane (MRBC). In addition, we check their biological activity based on antioxidant, anti-inflammatory, and hemolytic activity. We also present, on the basis of ultra-performance liquid chromatography (UPLC) analysis, the exact composition of the polyphenol composition of the tested extracts. Moreover, a completely new element of the proposed research is to measure the coefficient of dichroism and order parameter in the erythrocyte membrane modified with the extracts from BH and BS using the Fourier Transform Infrared Spectroscopy (FTIR) technique. Due to the lack of specialized, available literature referring to those measurements, our work is pioneering. For testing, we used the red blood cell (RBC), which is a very good research model, because it meets the basic functions assigned to the cell membrane (active and passive transport, creation of ion and electric gradients) and at the same time has a simplified structure compared to other cell membranes.

## Materials and Methods

### Reagent and Standard

Formic acid, methanol, AA (L(+) ascorbic acid), AAPH (2,2′-azobis(2-amidinopropane)hydrochloride), MC540 (merocyanine 540), and enzyme COX-2 (cyclo-oxygenase) were purchased from Sigma-Aldrich (Steinheim, Germany). Acetonitrile was purchased from Merck (Darmstadt, Germany). Quercetin-3-*O*-glucoside, quercetin 3-*O*-galactoside, quercetin-3-*O*-rutinoside, (+)catechin, (−)epicatechin, procyanidin B2-3-*O*-gallate, procyanidin B3, vitexin, and luteolin-3-*O*-glucoside were purchased from Extrasynthese (Lyon, France). The DPH-PA (3-(4-(6-phenyl)-1,3,5-hexatrienyl) phenylpropionic acid), Laurdan ((6-dodecanoyl-2-dimethylaminonaphthalene)), and DPH (1,6-diphenyl-1,3,5-hexatriene) fluorescence probes were purchased from Life Technologies (California, USA).

### Erythrocyte Cells and Erythrocyte Membranes

The investigation was conducted with erythrocytes (RBC) and their membranes (MRBC) obtained from fresh heparinated pig blood according to the method of Dodge et al. (1963). The choice of pig erythrocytes was dictated by the fact that this cell’s percentage content of lipids is closest to that of the human erythrocyte, and the blood was readily available. Erythrocytes are also a good research model due to its simplified structure compared to other cell membranes (they have only outer membrane, without internal organelles) and perform enough functions for being treated as a representative model.

Fresh blood was taken each time to a physiological solution of sodium chloride with heparin added. The content of erythrocyte membranes in the samples was determined on the basis of protein concentration, which was assayed using the Bradford method (1976), and it was 100 mg/ml.

### Plant Material

Buckwheat husk (100 g) and stalk were harvested from the Garden of Medicinal Plants herbarium of the Medical University in Wroclaw, Poland, by cultivation in the University’s experimental field. After harvest, the BH and BS were directly frozen in liquid nitrogen and freeze-dried (24 h; Alpha 1-4 LSC, Christ, Germany). The homogeneous powders were obtained by crushing the dried tissues using a closed laboratory mill to avoid hydration. Powders were kept in a refrigerator (−80 °C) until extract preparation.

### Extraction Procedure and the Content of Polyphenols

The extraction procedure of polyphenols was described previously by Gąsiorowski et al. (1997). Polyphenols were isolated from hulls and leaves by extraction with water containing 200 ppm SO2, the ratio of this solvent to leaves being 3:1 (v/v). The extract was adsorbed on Purolite AP 400 resin (UK) for further purification. The polyphenols were then eluted out with 80 % ethanol, concentrated, and freeze-dried. The content of polyphenols in individual preparations was determined by means of UPLC/DAD and the UPLC/ESI/MS method of analyses described earlier by Oszmiański et al. (2011).

### Hemolytic Activity and Osmotic Resistance

The hemolytic method was described earlier by Cyboran et al. (2015). Buckwheat extracts were added in the concentration: 0.01, 0.025, 0.05, 0.075, and 0.1 mg ml^−1^. The extent of hemolysis was measured at 2 % hematocrit. The supernatant was assayed for hemoglobin content using an UV–Vis spectrophotometer (Specord 40, AnalytikJena) at 540 nm wavelength. Hemoglobin concentration in the supernatant, expressed as percentage of hemoglobin concentration in the supernatant of totally hemolyzed cells, was assumed as the measure of the extent of hemolysis.

The osmotic resistance was investigated using the spectrophotometric method described in our earlier work (Włoch et al. 2013) with minor modification. A red blood cell (at 2 % hematocrit) suspension containing BH and BS extracts of 0.05 mg ml^−1^ concentration was prepared. On the basis of the results obtained, the relation was determined between the percentage of hemolysis and NaCl concentration in the solution. Next, using the obtained plots, the NaCl percent concentrations that caused 50 % hemolysis (*C*_50_) were found. The *C*_50_ values were taken as the measure of osmotic resistance. If a determined sodium chloride concentration is higher than that of control cells, the osmotic resistance of the erythrocytes is regarded as lower, and vice versa.

### Shape of Erythrocytes

The shape of red blood cells modified with BH and BS extracts was investigated using the optical and scanning electron microscopes. The methods were described earlier in Włoch et al. (2013).

For investigation with the optical microscope, the red cells contained 0.01 and 0.1 mg ml^−1^, and with the scanning electron microscope the concentration of the BH and BS extracts was 0.05 mg ml^−1^. The individual forms of erythrocyte cells were ascribed morphological indices according to the Bessis and Brecht scale (Deuticke 2003), which for stomatocytes assume negative values from −1 to −4 and for echinocytes from 1 to 4.

### Fluidity and Packing Arrangement of the Membrane

In order to determine the likely location of the polyphenolic compounds in the membrane and examine their impact on physical parameters of erythrocyte membrane, the fluorimetric and infrared spectroscopy method was applied. The dichroism method was innovatively used for the erythrocyte membrane.

#### Fluorimetric Methods

 The fluorescent probes Laurdan, DPH, and MC540 were used to monitor the effects at different bilayer depths, and to obtain additional information about the location of the components of BS and BH extracts in the erythrocyte membrane. Merocyanine 540 (MC 540) is a probe adsorbing at the membrane surface. Its negative charge is localized near the membrane bilayer interface, just above the glycerol skeleton (Alay et al. 2003). Packing density in the hydrophilic part of lipids of the erythrocyte membrane was studied using the Laurdan fluorescence probe, using method described earlier by Włoch et al. (2013) with minor modification. DPH probe is supposed to distribute in the hydrophobic part of the bilayer. BH and BS extracts were added at concentrations ranging from 0.01 to 0.1 mg ml^−1^. The excitation and emission wavelengths were as follows: for DPH, $\lambda_{\text{exc}}$ = 360 nm and *λ*_em_ = 425 nm; for Laurdan, $\lambda_{\text{exc}}$ = 360 nm and the emission wavelengths were *λ*_em_ = 440 nm and *λ*_em_ = 490 nm; and for MC540, $\lambda_{\text{exc}}$ = 540 nm and *λ*_em_ = 590 nm.

Packing density in the hydrophilic part of lipids of the erythrocyte membrane was determined on the basis of generalized polarization (GP) of Laurdan, calculated with the formula (Parasassi et al. 1998):

1 $${\text{GP}} = \frac{{I_{\text{b}} - I_{\text{r}} }}{{I_{\text{b}} + I_{\text{r}} }},$$

where $I_{\text{b}}$ is fluorescence intensity at $\lambda$ = 440 nm and $I_{\text{r}}$ is fluorescence intensity at $\lambda$ = 490 nm.

Fluorescence anisotropy (*A*) for DPH probe was calculated using the formula (Lakowicz 2006):

2 $$A = \frac{{I_{\text{II}} - GI_{ \bot } }}{{I_{\text{II}} + GI_{ \bot } }},$$

where *I*_II_ and *I*_⊥_—fluorescence intensities observed in directions parallel and perpendicular to the polarization direction of the exciting wave, respectively. *G* is an apparatus constant dependent on emission wavelength.

#### Fourier Transform Infrared Spectroscopy studies

The FTIR experiments were prepared as follows: red blood cell membrane (MRBC) was washed three times in 0.9 % NaCl solution; next, the ghost suspension was incubated (600 µl ghosts + 600 µl physiological salt or physiological salt with the extracts 0.1 at mg ml^−1^ concentration) for 24 h at 37 °C. After incubation, the samples were centrifuged for 15 min at 30,000×*g*, and 50 μl condensed membrane suspension was applied on the ZnSn plate. The plate coated with the ghost layer was then placed for 24 h in a vacuum to remove the water. To rehydrate the film, 100 μl D_2_O or H_2_O was added on the sample surface and film was incubated for 24 h in 4 °C. The measurements of the dry and rehydrated films were performed using a Thermo Nicolet 6700 MCT (Thermo Fisher Scientific, Waltham, MA), and each single spectrum was obtained from 128 records at 2 cm^−1^ resolution in the range 700–4000 cm^−1^. For each film, the measurements were made using polarized and unpolarized light. Preliminary elaboration of a spectrum was done using the EZ OMNIC v 8.0 program, also of the Thermo Nicolet make. After filtering the noise out from the spectrum of the object studied, the spectrum of the D_2_O or H_2_O was subtracted in order to remove a strong band of water and the baseline was corrected.

##### Fluidity and Hydration of Membrane

In unpolarized spectra thus prepared, we examined four bands associated with lipids located in the range 3000–2800 cm^−1^ from vibrations of CH_2_ and CH_3_ groups of alkyl chains, in 1760–1700 cm^−1^, which correspond to carbonyl group (C=O) vibrations, 1360–1200 cm^−1^ corresponding phosphate group (PO_2_^−^) vibrations, and about 972 cm^−1^—trimethyl ammonium group vibrations. We also investigated the I amide band (1700–1600 cm^−1^) that are generally employed to study protein secondary structures. Principal amide I frequencies of protein are β-sheet strong (1612–1640 cm^−1^), random (1645 cm^−1^), α—helix (1650–1657 cm^−1^), and β-sheet weak (1670–1690 cm^−1^) (Pelton and McLean 2000).

The frequency of methylene and methyl groups of alkyl chains depend on mobility (fluidity) of the chains and increase, e.g., with increasing temperature or during transition from the gel state to the liquid crystalline state. The increase in wavenumber of these bands testifies to an increase of liquidity of the hydrophobic part of the membrane.

The carbonyl group and even more the phosphate groups form hydrogen bonds with water. The carbonyl group can bind one molecule of water, while the phosphate group can bind a few. Hence the carbonyl and phosphate bands of phospholipids are the sum of the vibrations of C=O or PO_2_ groups that are at different degrees of hydration (Attar et al. 2000; Lewis et al. 1994). Vibrations of C=O and PO_2_ groups which do not have water bonds are represented by the wavenumbers ≈1742 and ≈1253 cm^−1^, respectively. Each bound water molecule moves these values by about 20 cm^−1^ in the direction of smaller values. The changes observed in these bands testify, therefore, to changes in the degree of hydration of the carbonyl and phosphate groups. Similar changes are also observed when the membrane embeds the compounds having in their structure OH groups which may also form hydrogen bonds with the carbonyl groups or phosphoric lipids.

##### Infrared Linear Dichroism Spectroscopy

Polarized infrared spectroscopy has proven in the past to be a powerful tool for the determination of the orientation and conformation of specific segments within macroscopically aligned bilayers (Binder 2003; Okamura et al. 1990). We used this technique to determine the effects of BH and BS extracts on the conformation of MRBC. For polarized ATR measurements, we used an ZnSn ATR 10-reflective crystal, face angle *υ* = 45^0^. A grid polarizer on KRS5 was placed behind the ATR unit, and the settings for parallel and perpendicular polarized light were under manual control.

Infrared spectra of the solid and hydrated film were recorded with parallel and perpendicular polarized light, using parallel and perpendicular polarized spectra of the blank ZnSn plate as single-beam references. These spectra were used to calculate the dichroic ratio R of a particular absorption band. R is defined as the ratio of the integrated absorption intensity of the infrared radiation polarized parallel (*A*_II_) to that polarized perpendicular (*A*_⊥_) to the plane of incidence. The plane of incidence is determined by the incoming and the reflected IR beam (Binder 2003; Hübner and Mantsch 1991).

3 $$R = \frac{{A_{\text{II}} }}{{A_{ \bot } }}$$

Based on the dichroic ratio *R*, is determined the order parameter *S*_IR_, and connected with them the mean tilt angle *β* of the transition moment of the active group, with respect to the normal of the ATR crystal plane. For the polar head group (i.e., C=O double-bond vibration, PO_2_^−^ vibrations, CO–O single-bond vibration, and N–CH_3_ vibration), we used the Eq. (4):

4 $$S_{\text{IR}} = \frac{R - 2}{R + 1,45}$$

or

5 $$S_{\text{IR}} = - 2\frac{R - 2}{R + 1,45}$$

for the order parameter of the lipid chain (CH_2_ stretching vibrations). The mean tilt angle (*θ*) is determined from the formula (6):

6 $$\theta \, \approx \arccos \left( {\sqrt {\frac{1}{3}\left( {2S + 1} \right)} } \right).$$

### Biological Activity

Biological activity of the studied extracts (BS, BH) was assessed on the basis of their antioxidant and anti-inflammatory activity.

#### Antioxidant Activity

The antioxidant activity of the extracts was determined using the spectrophotometric and fluorimetric methods described in our earlier work (Włoch et al. 2013) with minor modification. In the first method, the oxidizing agent was UVC radiation and in the second method AAPH oxidation inducer. The extent of lipid oxidation inhibition in the spectrophotometric method was expressed as a percentage calculated from the formula (7):

7 $${\text{Inhibition }}\left( \% \right) \, = \frac{{(A_{0} - A)}}{{A_{0} }} \times 100\;\%,$$

where *A*_0_ is absorbance of control sample and *A* is absorbance of sample with extract.

The percentage of lipid oxidation inhibition in fluorimetric method was calculated from the following formula (8):

8 $${\text{Inhibition }}\left( \% \right) \, = \frac{{(F_{\text{x}} - F_{\text{u}} )}}{{(F_{\text{k}} - F_{\text{u}} )}} \times 100\;\%,$$

where *F*_X_ is relative fluorescence of an UVC-irradiated sample, or oxidized by AAPH for 30 min in the presence of extracts, *F*_U_ is relative fluorescence of control sample, oxidized by AAPH or UVC radiation, measured after 30 min, and *F*_K_ is relative fluorescence of the blank sample, not subjected to oxidation procedures, measured after 30 min.

In both used methods, the antioxidant activity was determined as the concentration at which lipid peroxidation was inhibited by 50 %. Antioxidant activity of the extract was compared with activity of L(+) ascorbic acid (AA)—the standard antioxidant.

#### Anti-inflammatory Activity

The anti-inflammatory activity of the substances, established on the basis of the modified method given in the work by Cyboran et al. (2015), was assayed with a spectrophotometric measurement of inhibition of activity of the cyclo-oxygenase (COX-2). Percentage of inhibition was calculated using the following formula (9):

9 $${\text{Inhibition }}\left( \% \right) \, = \frac{{\Delta A_{\text{control}} - \Delta A_{\text{sample}} }}{{\Delta A_{\text{control}} }} \times 100\;\%,$$

where Δ*A*_control_ and Δ*A*_sample_ denote the increase of absorbance after 3 min from substrate addition to the probe without and with substances tested, respectively.

### Statistical Analyses

Data are shown as mean values ± standard deviation (*n* = 6). These data were compared using Duncan’s multiple range test. Data were analyzed by one-way analysis of variance (ANOVA) using Statistica 12.5 (StatSoft PL). Differences were considered statistically significant at *p* < 0.05.

## Results

### The Contents of Polyphenols

The results of phenolic compound analysis of the BH and BS extracts by RP-UPLC-ESITOF-MS are shown in Table 1 (and Suppl. 1–5). Twenty-two flavonoids and two phenolic acid derivatives were characterized and quantified. These are presented in Table 1, with their retention times, MS^−^ ion *m*/*z*, MS fragments, *λ*_max_, and contents. To identify compounds for which no commercial standards were available, we checked the generated molecular formula obtained by TOF analysis and also studied their respective fragments. The ion found at *m*/*z* 341.0872 and its fragments at *m*/*z* 251.1121 and 179. 0339 was identified as caffeic acid hexose. Two compounds, 1 and 4, were detected as caffeic acid hexose in buckwheat extracts at a retention time of 2.23 and 3.59 min, respectively.

**Table 1:** Phenolic compounds determined by UPLC-ESI-TOF–MS in an extract of buckwheat husk and stalk

Compounds	Content husk (mg g^−1^)	Content stalk (mg g^−1^)	Rt (min)	[M-H] (*m*/*z*)	MS–MS	*λ*_max_ (nm)
Caffeic acid hexose	0.92	0.02	2.23	341.0872	251.1121/179.0339	323
Procyanidin B3	14.14	0.56	2.78	577.1349	289.0709	280
(+)Catechin glucoside	3.00	0.77	3.74	451.1245	289.0705/245.0814	280
Caffeic acid hexose	5.55	1.47	3.59	341.0872	251.1121/179.0339	323
(+)Catechin	4.01	0.99	4.93	289.0712	245.0818	280
(−)Epicatechin glucoside	9.10	3.43	4.03	451.1245	289.0705/245.0814	280
1-O-Caffeoyl-6-O-alpharhamnopyranosyl-betaglycopyranoside	16.02	1.06	4.16	487.1450	451.1250/179.0345/135.0450	314
(−)Epicatechin	7.73	1.06	4.93	289.0712	245.0818	280
(Epi)afzelchine(epi)catechin isomer A	1.16	1.45	5.18	561.1390	543.1289/435.1066/425.0865/289.0710/271.0605	280
Procyanidin B2-3-*O*-gallate	3.15	0.54	5.39	729.1448	577.1359/407.0765/289.0708	280
Orientin	1.83	0.29	6.40	447.0932	357.0622/327.0512/297.0396	334
Isorientin	13.00	22.44	6.47	447.0932	357.0622/327.0512/297.0396	334
(−)Epicatechin gallate	3.60	1.35	6.89	441.0821	289.0709/169.0165	280
Rutin	21.63	10.30	7.07	609.1464	300.0269	360
Vitexin	23.82	83.08	7.28	431.0983	311.0553/283.0594	334
Heperin	83.20	162.01	7.28	463.0877	300.0269/271.0618	463
Rutin isomer	51.86	89.14	7.38	609.1464	300.0269	360
Isoquercitrin	49.04	93.31	7.54	463.0877	300.0269/271.0618	463
Isovitexin	25.47	54.69	7.54	431.0983	311.0553/283.0594	334
Epiafzelchineepicatechin-*O*-methyl gallate	9.50	14.80	7.81	727.1658	561.1357/289.0710/271.0605	280
Luteolin glycoside	2.53	1.03	8.37	447.1034	284.0562	348
ProcyanidinB2 dimethyl gallate	0.37	0.35	9.26	757.1755	605.1243/289.0731	280
Quercitrin	13.81	1.03	9.74	447.0930	300.0269/179.0264	463
Epiafzelchineepicatechin-*O*-di-methylgallate	7.47	1.96	10.00	741.1820	469.1129/319.0818/271.0603	280
Total	371.91	547.13				

Compound 2 at tr. 2.78 showed a mass spectrum typical for B-type procyanidin dimers with an [M-H]^−^ ion at *m*/*z* 577.1349 as the base peak. The ion at *m*/*z* 289.0709 was derived from the cleavage of the link between the two procyanidin monomers through the fragmentation (Gu et al. 2003). The compound was identified as procyanidin B3 with the aid of a reference compound. Compounds 5 and 8 had similar mass spectra and λ_max_ and were tentatively identified as (+) catechin and (−) epicatechin, respectively, according to their retention time compared with respective standards. Two catechin glucosides were detected in this study (Suppl. 2). Compounds 3 and 6 with mass 451.1245 showed a fragment ion at *m*/*z* 289.0705 corresponding to the breaking of the catechin glucoside linkage. The present data are in agreement with Zheng et al. (2008). The peak number 7 in negative mode at 487.1450 *m*/*z* (Suppl. 3), with the fragment obtained for this compound at *m*/*z* 179.0345, corresponds to caffeic acid. This compound was identified as 1-*O*-caffeoyl-6-*O*-alpha-rhamnopyranosyl-beta-glycopyranoside (swertiamacroside) according to Wang et al. (2009). The (epi) afzelchine(epi)catechin dimer isomers of propelargonidins were detected in the buckwheat extract according to Gu et al. (2003), and compound 9 was identified in negative mode at *m*/*z* 561.1390 and its fragments (epi) catechin at *m*/*z* 289.0710 and (epi)afzelchine at *m*/*z* 271.0605.

According to the mass spectra, the galloylated flavanols were detected in the buckwheat samples: epicatechin gallate (peak 13) (441.0821/289.0709) and procyanidin B2-3-O-gallate (peak 10) (729.1448/289.0708). Their presence in buckwheat was reported by Quettier-Deleu et al. (2000). Acylated propelargonidins and proanthocyanidins were found in both the buckwheat extracts (Suppl. 4). These compounds 20, 22, and 24, epiafzelchineepicatechin-*O*-methyl gallate, procyanidin B2 dimethylgallate, and epiafzelchineepicatechin-O-di methyl gallate, were identified according to the mass spectra shown at *m*/*z* 727.1658, 757.1755, and 741.1820, respectively.

These compounds were found in buckwheat also by Ölshläger et al. (2008). Five flavones and five flavonols were identified in the buckwheat samples (Suppl. 5). Compounds 11, 12, 15, 19, and 21 presented signals at *m*/*z* 447.0932, 431.0984, 431.0983, and 447.1034, corresponding to orientin, isorientin, vitexin, isovitexin, and luteolin glucoside, respectively. The UV absorbance, mass spectra and fragmentation mass data were compared with the standards and data obtained by March et al. (2006). Flavonol compounds 14, 16, 17, 18, and 23 were identified on the basis of the measured *m*/*z*, fragment ions, and UV absorbance, and by spiking with the commercial standards. Two compounds 14 and 17 showing molecular ions at 609.1464 and fragment ion 300.0269 *m*/*z* corresponding to the loss of the rutinose unit were identified as rutin and isomer of rutin (Suppl. 5 *m*/*z* 609), respectively. Compounds 16 and 18 showed the same molecular ion at 463.0877 *m*/*z* and a fragment at 300.0269 and 271.0618. After comparison of retention time and UV spectrum with standards, they were identified as heperin and isoquercitrin, respectively. These compounds were described by He et al. (2008a) and Hvattum and Ekeberg (2003). Compounds 23 with molecular ion 447.0930 and the fragments at *m*/*z* 300.0269 and 179.0264 indicated the presence of quercitrin. The same fragmentation pattern was reported by Hvattum and Ekeberg (2003). Compound 21, with molecular ion 447.1034, fragmentation 284.0562, and retention time at 8.37 min, corresponds to luteolin glycoside standard. These compounds were found in buckwheat also by Ölshläger et al. (2008). Five flavones and five flavonols were identified in the buckwheat samples (Suppl. 5).

Favan-3-ols (proanthocyanidin and catechin derivatives) and phenolic acid derivatives were the more abounded phenolic groups found in BH extract than in BS extract, and they constituted 107.35 and 40.11 mg/g of powder, respectively. However, the BS extract contained much more total of flavonols and flavones than BH extract, 517.32 and 286.19 mg/g of powder, respectively.

### Hemolytic Activity and Osmotic Resistance

In the experiment, we examined whether buckwheat extracts induce hemolysis of red blood cells in the concentration: 0.01, 0.025, 0.05, 0.075, and 0.1 mg ml^−1^. The results showed that the percentage of hemolysis was at a similar level as for control blood cells and did not exceed 2 %. These studies showed that the tested extracts not only do not cause hemolysis, but also protect the red blood cells against it. These results are also consistent with the osmotic resistance tests.

**Fig. 1:**
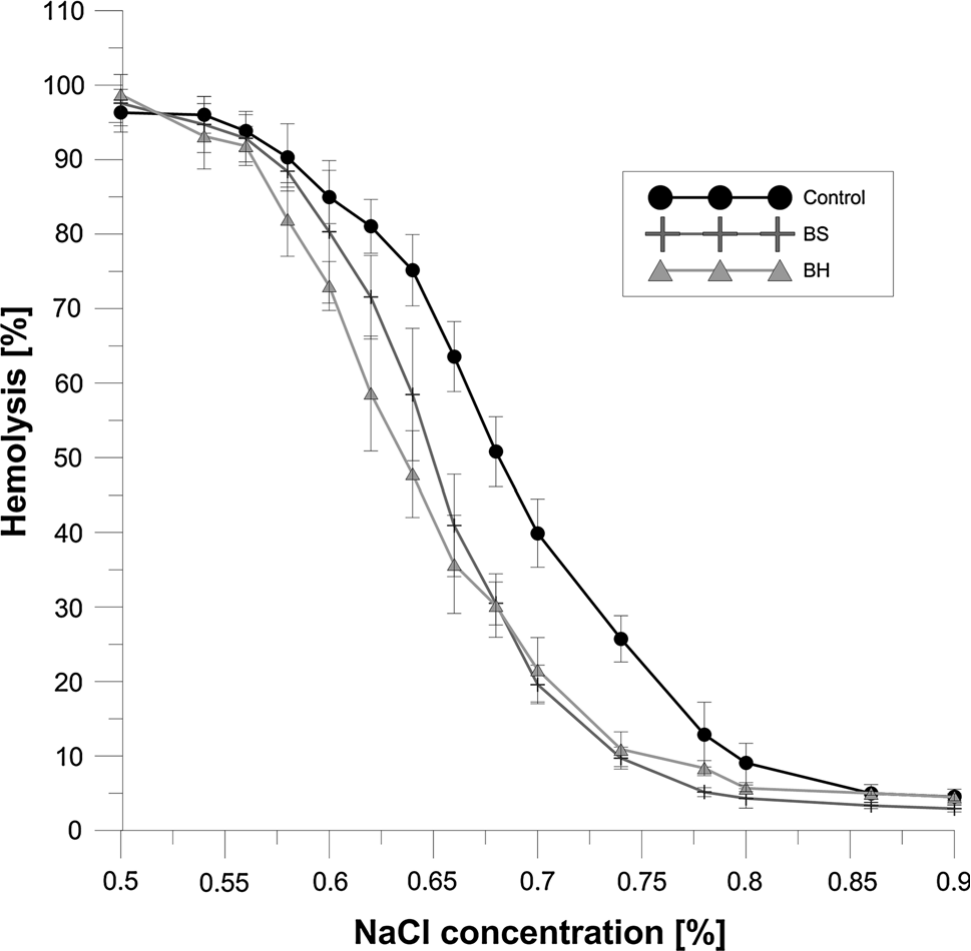
Percentage of hemolysis of cells modified with BH and BS extracts at 0.05 mg ml^−1^ concentration versus sodium chloride concentration

The effect on osmotic resistance was examined for BH and BS extracts used in a concentration of 0.05 mg ml^−1^. This impact was established on the basis of the comparison of hemolytic curves for blood cells modified with the compounds with unmodified, control cells. Figure 1 shows the relationship between percentage of modified blood cell hemolysis (BS and BH) and unmodified, and percent concentration of NaCl. From the graph, it appears that the extract-modified cells from the outset markedly move the hemolytic curves in the direction of lower NaCl concentrations when compared with the control. Hemolytic curve shifting toward lower NaCl concentrations testifies to an increase in the osmotic resistance of erythrocytes. On the basis of the hemolytic curves, the concentration *C*_50_ was determined, i.e., the concentration at which 50 % of the erythrocytes had been hemolysed. The *C*_50_ was 0.64 and 0.65 for BH and BS, respectively. Comparing both the extracts, we see that their effect on hemolysis is roughly the same.

### Shape of Erythrocytes

**Fig. 2:**
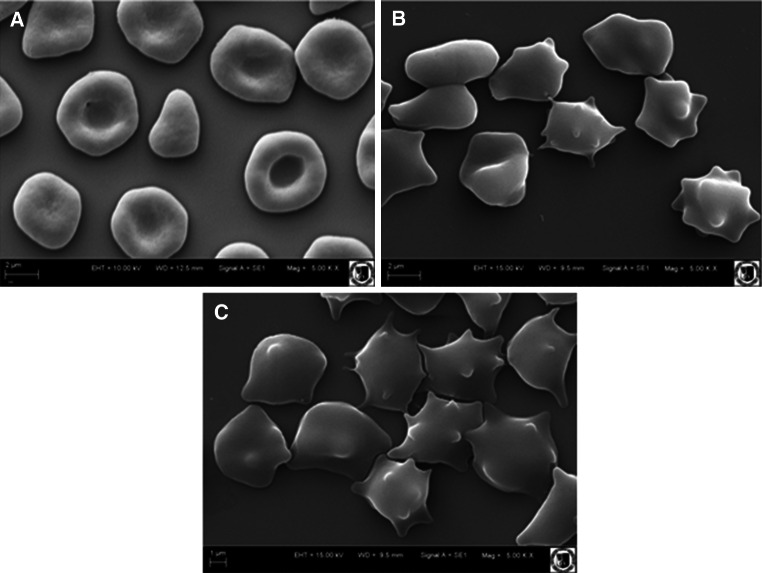
Effects of BS and BH extracts on the morphology of erythrocytes observed with the electron microscope, at 0.05 mg ml^−1^ concentration. **a** Control (unmodified erythrocytes), **b** modified with BH, and **c** modified with BS

Examination of erythrocyte shape changes induced by the extracts BH and BS showed that blood cells are changing shape under the influence of the extracts from the discocyte (doubly concaved) to the echinocyte (bubbles with a number of tabs) (Fig. 2). The size of these changes depends on the concentration of the extract; the higher the concentration, the more echinocytes are formed (Bonarska-Kujawa et al. 2014; Suwalsky et al. 2008; Suwalsky and Avello 2014;), and a larger percentage of these forms is apparent in the case of erythrocytes modified with BS extract. Table 2 shows the percentage of erythrocyte shapes when modified with BH and BS extracts at concentrations of 0.1 and 1.0 mg ml^−1^. The results indicate that BH and BS extracts are able to penetrate the erythrocyte membrane, causing a change in the erythrocyte shape.

**Table 2:** Mean percentage of erythrocyte shapes formed in the presence of BH and BS extracts applied at 0.1 and 1.0 mg ml^−1^

Average percent share of individual forms of erythrocytes ± SD					
	Control	BH extract		BS extract	
Shape of erythrocytes	–	0.1 mg ml^−1^	1 mg ml^−1^	0.1 mg ml^−1^	1 mg ml^−1^
Spherostomatocytes (−4)	0	0	0	0	0
Stomatocytes II (−3)	0	0	0	0	0
Stomatocytes I (−2)	0	0	0	0	0
Discostomatocytes (−1)	4.90 ± 0.30	13.03 ± 0.50	0.40 ± 0.21	0	0
Discocytes (0)	81.44 ± 0.26	72.93 ± 0.31	40.00 ± 0.32	11.49 ± 0.24	12.41 ± 0.41
Discoechinocytes (1)	13.66 ± 0.19	11.78 ± 0.34	52.50 ± 0.38	24.19 ± 0.39	10.52 ± 0.59
Echinocytes (2)	0	2.26 ± 0.36	6.74 ± 0.37	64.32 ± 0.31	56.21 ± 0.44
Spheroechinocytes (3)	0	0	0.36 ± 0.19	0	20.86 ± 0.50
Spherocytes (4)	0	0	0	0	0

### Fluidity and Packing Arrangement of the Membrane

#### Fluorimetric Method

Using the fluorimetric method and 3 probes (MC540, Laurdan and DPH) that embed at different depths, changes that produce the extracts BH and BS in the MRBC were studied. For each of the extract, the experiments were conducted with five concentrations in a non-lytic range: from 0.01 to 0.1 mg ml^−1^, at 37 °C.

**Fig. 3:**
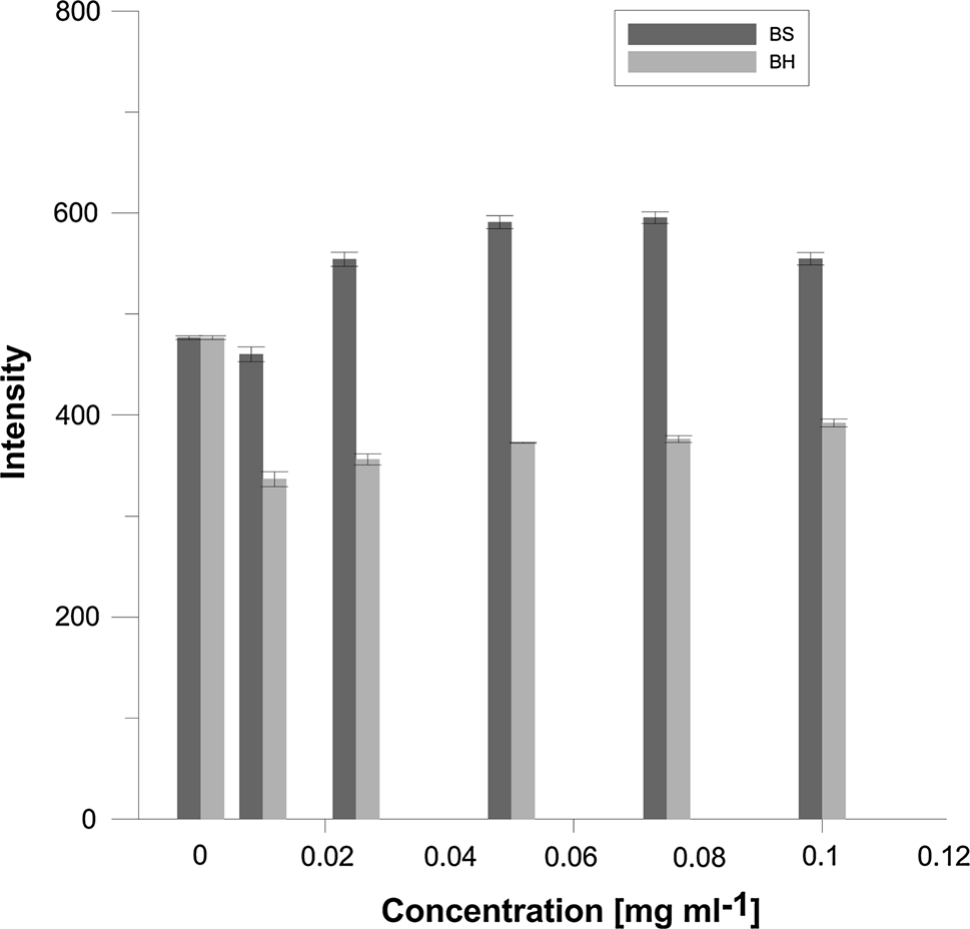
Changes of intensity of MC 540 probe for erythrocytes modified with BS and BH extracts

The first probe, MC540, adsorbs on the surface of the membrane, slightly above the glycerol skeleton. In aqueous environment, this probe emits little light, and in hydrophobic environment its intensity increases significantly. Studies have shown that the differences in intensity of fluorescence arise also from differences in the probe’s adsorption level to the bilayer that can have varying degrees of packing order (Fig. 3). Therefore, its fluorescence should reflect the degree of order of lipids in a phospholipid bilayer. The greater the fluorescence, the lesser the packing order.

The second probe, Laurdan, locates in the hydrophilic–hydrophobic interface of the bilayer, and its fluorophore takes position near the phospholipid glycerol groups and is sensitive to polarity changes and dynamic properties at the membrane lipid–water interface (Parasassi et al. 1991, 1998). Studies have shown that the extracts cause a significant decrease in GP with increasing extract concentration (Table 3), BS extract showing greater changes. The decrease of the GP coefficient indicates that more water molecules are incorporated at the level of the glycerol backbone. These results suggest that the extracts cause large changes in the polar group region.

**Table 3:** Values of fluorescence anisotropy (A) of DPH probe and values of generalized polarization (GP) of the Laurdan probe for MRBC modified by BS and BH at 37 °C

Extract	BS		BH	
Concentration (mg ml^−1^)	GP ± SD	A ± SD	GP ± SD	A ± SD
Control	0.426 ± 0.032^a^	0.260 ± 0.003^a^	0.426 ± 0.032^a^	0.260 ± 0.003^c^
0.01	0.393 ± 0.011^a^	0.263 ± 0.002^a^	0.408 ± 0.008^a^	0.267 ± 0.006^c^
0.025	0.358 ± 0.026^b^	0.264 ± 0.004^a^	0.366 ± 0.028^b^	0.265 ± 0.005^c^
0.05	0.323 ± 0.024^b^	0.262 ± 0.009^a^	0.346 ± 0.034^b^	0.276 ± 0.001^b^
0.075	0.297 ± 0.059^bc^	0.262 ± 0.002^a^	0.323 ± 0.031^bc^	0.280 ± 0.009^ab^
0.1	0.225 ± 0.054^d^	0.265 ± 0.003^a^	0.254 ± 0.034^d^	0.284 ± 0.003^a^

Statistically significant differences between results of control and modified samples are denoted

Different letters (a–d) within the same column indicate significant differences at *p* < 0.05 by Duncan’s test

The third probe, DPH, takes position deep in the hydrophobic area. Using changes of fluorescence anisotropy of the DPH probe, the impact of BS and BH on fluidity of the erythrocyte membrane was studied. The results show a slight increase in fluorescence anisotropy with increasing concentration of extract (Table 3) for BH. The results obtained indicate that the extracts induce increased order in the hydrophobic area (within the hydrocarbon chains).

#### FTIR Studies

##### Fluidity and Hydration of Membrane

MRBC containing many different components, aside from interacting with water molecules via hydrogen bonds, interact also with other membrane components. These interactions can be stronger than those with water molecules, so that we observe summarized bands whose frequency maxima depend on hydration of the membranes and interactions with additional compounds which can appear in the membranes.

The carbonyl band of MRBC is a sum of the components coming from various phospholipids of the membrane that form (or do not form) various hydrogen bonds. The phosphate band of the erythrocyte membrane summarizes absorption of all the different membrane phospholipids that occur in all possible hydration states. An increase in the wavenumber of the maximum of this band is indicative of the location of the lipid phosphate polar headgroup in less polar environments and a decrease in headgroup hydration (or hydrogen bonding) (Lewis et al. 1996; Lewis and McElhaney 2000; Żyłka et al. 2009). The highest frequency component refers to asymmetric stretching vibrations that are free of hydrogen bonds, while each water molecule that forms a hydrogen bond with the phosphate group diminishes the corresponding wavenumber by 20 units. The degree of hydration of individual groups can be calculated as the ratio of the surface intensity of absorbance of the groups (CO or PO_2_^−^) having hydrogen bonds to surface intensity of the full band.

**Fig. 4:**
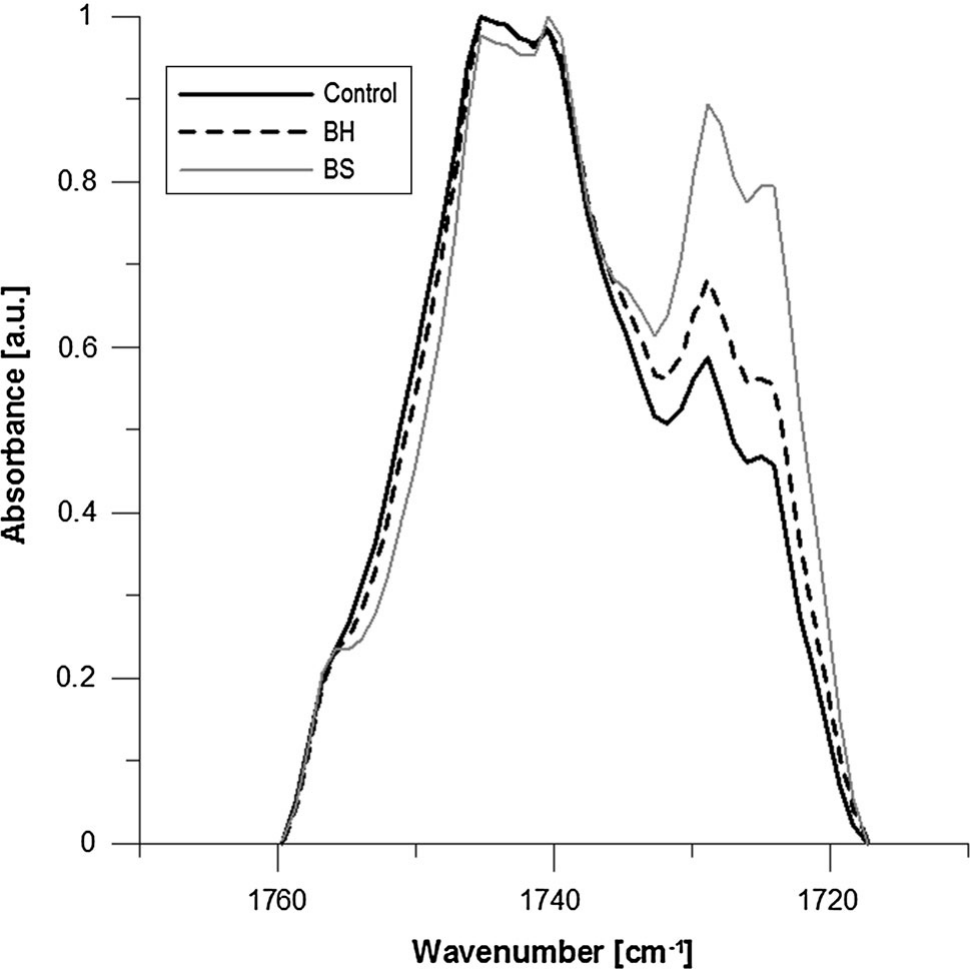
Carbonyl band. The maximum at 1742 cm^−1^ represents vibration region of C=O groups “free” of water molecules, while the second maximum at 1727 cm^−1^ comes from C=O groups having hydrogen bonds

**Fig. 5:**
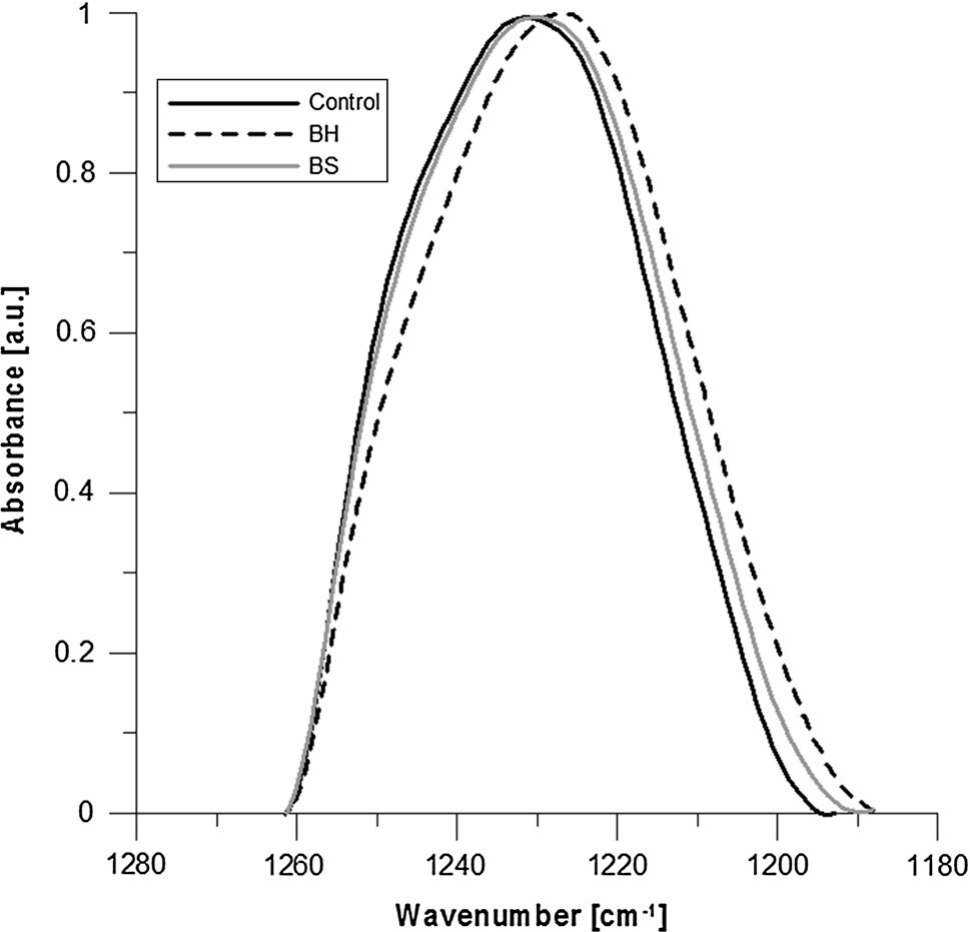
Phosphate band I. A shift of phosphate band toward lower frequencies after addition of BH and BS extract is visible, which confirms binding of water molecules

Figure 4 shows the increase in hydration in carbonyl band for both test compounds. However, a greater hydration was observed for ghosts with BS added that was 8.2 and 3.3 % for BH compared with control. In the case of the phosphate band, an increase in hydration level was observed in both cases of the tested compounds too, but a greater one for ghosts with extract added—4.2 % for BH and 3.6 % for BS compared to control (Fig. 5).

Comparing the spectra obtained for MRBC and treated with the compounds, significant changes were observed in the carbonyl band (in particular for BS extract—Fig. 4), and much more subtle in the case of the phosphate group bands.

##### Infrared Linear Dichroism Spectroscopy

This method makes possible a determination of the orientation and conformation of specific segments of lipid molecules within aligned bilayers (Fringeli 1977; Hübner and Mantsch 1991; Müller et al. 1996; Okamura et al. 1990). From the dichroic ratios *R*_ATR_ of the CH_2_ stretching vibrational bands, the inclination of the acyl chains of the phospholipid can be determined. From the *R*_ATR_ values of the choline, phosphate, and carboxylate bands, it should be possible to obtain information on the state and the orientation of the head groups of the lipids. Using the formulae given in the method description, the dichroism coefficients (*R*_ATR_) were calculated and then the order parameters (*S*_IR_) and inclination angles (*θ*) of respective groups in the membrane for MRBC and MRBC modified with the extracts. The results are given in Table 4.

**Table 4:** *R*_ATR_, *S*_IR_, and *θ* values for MRBC (control) and MRBC with extract mixtures

	Wavenumbers (in cm^−1^)	*R*_ATR_	*S*_IR_	*θ*°
MRBC	2919	1.318	0.353	41.072
MRBC + BH		1.240	0.401	39.191
MRBC + BS	(CH_2_)as	1.248	0.396	39.382
MRBC	2850	1.143	0.464	36.702
MRBC + BH		1.065	0.518	34.547
MRBC + BS	(CH_2_)s	1.088	0.501	35.215
MRBC	1730	1.115	−0.241	65.465
MRBC + BH		0.933	−0.306	68.935
MRBC + BS	C=O	1.125	−0.238	65.301
MRBC	1250	1.200	−0.213	64.077
MRBC + BH		1.405	−0.150	61.130
MRBC + BS	PO2-as	1.359	−0.164	61.746
MRBC	970	1.250	−0.197	63.310
MRBC + BH		1.750	−0.058	57.129
MRBC + BS	N–C ip	1.500	−0.123	59.932
MRBC	1650–1657	3.841	0.288	43.546
MRBC + BH		1.591	−0.099	58.853
MRBC + BS	α-helix	1.127	−0.237	65.264
MRBC	1645	2.267	0.055	52.521
MRBC + BH		3.167	0.204	46.755
MRBC + BS	Random	1.614	−0.093	58.590
MRBC	1612–1640	1.723	−0.065	57.411
MRBC + BH		1.523	−0.117	59.654
MRBC + BS	β-sheet	1.839	−0.037	56.241
MRBC	1670–1690	1.689	−0.073	57.772
MRBC + BH		1.125	−0.238	65.301
MRBC + BS	β-sheet	1.963	−0.008	55.073

Spectra were recorded with 0° or 90° polarized light (*T* = 23 °C, thick films on a ZnSe-crystal)

On the basis of the results obtained, it can be concluded that the addition of extracts to the MRBC causes a slight increase in the order parameter (*S*_IR_) in the acyl chains of lipids, which could mean a slight increase in order when compared to control. The angle of the axis of the chain decreases in relation to the control and chains set perpendicular to the plane. Bigger changes were observed after addition of BH extract.

In the case of phosphate of I and choline bands (the polar area of the lipid bilayer), the parameter S_IR_ assumed negative values. This means that the head is parallel to the plane. After adding the extracts, the values grow in relation to the control, and greater growth (both bands) was observed after addition of BH extract. These results indicate an increase in the disorder, while the angle of the lipid head axis decreases in relation to the control.

From the amide band I, the order coefficient (*S*_IR_) was also determined for parts corresponding to protein structures. On the basis of the results obtained, which are provided in Table 4, the impact of the extracts on the S_IR_ of proteins was found. The amide band I is the sum of all the proteins in the membrane, which is why discussion of the results obtained is not possible.

### Biological Activity

#### Antioxidant Activity

**Fig. 6:**
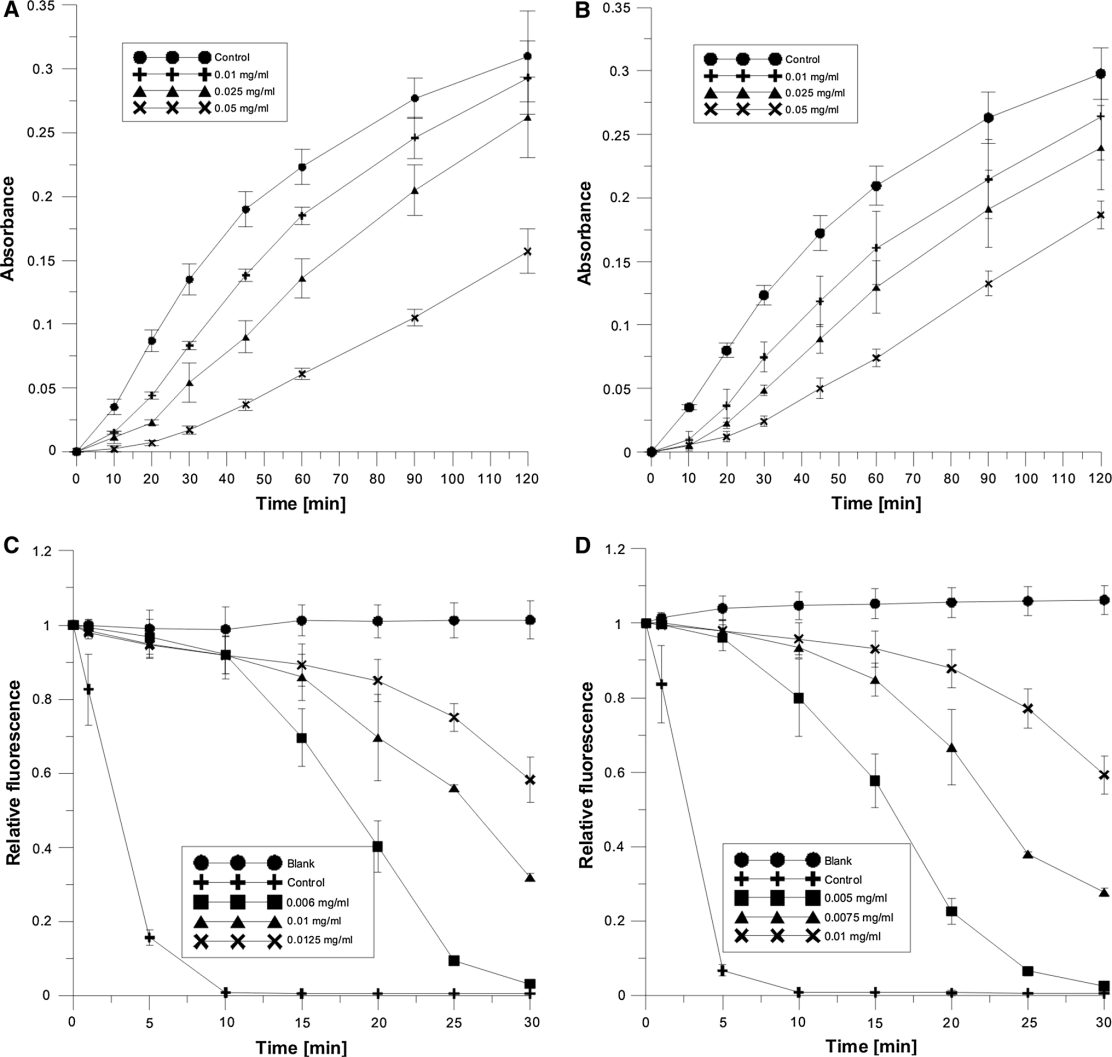
Kinetics of erythrocyte membrane oxidation caused by UVC radiation: **a** in the presence of BS extract; **b** in the presence of BH extract and by the compound AAPH; **c** in the presence of BS extract; and **d** in the presence of BH extract

The antioxidant activity of the extracts was determined using spectrophotometric and fluorimetric methods. With spectrophotometric method, the antioxidant activity of BH and BS was evaluated based on their ability to inhibit oxidation of erythrocyte membrane lipids induced by UVC radiation. The oxidation of lipids resulted in the creation of malondialdehyde (MDA), which reacted with thiobarbituric acid (TBA) producing a colorful product. The concentration of MDA was a measure of the antioxidant activity of BS and BH. The less the created MDA (also less the colorful adduct), the higher the activity of the extract. Figure 6a and b shows the kinetics of oxidation of erythrocyte lipids induced by UVC in 2-h time (for three concentrations). The curves show a relation between absorbance, proportional to the degree of oxidation, and time of erythrocyte ghost UVC irradiation. From the figure, it follows that with increasing irradiation time the absorption, which is a measure of lipid oxidation, increases. Based on the oxidation kinetics, for the oxidation time of 60 min, the percentage of oxidation inhibition was calculated and from the plot of the percentage inhibition versus extract concentration, the IC_50_ value was found, i.e., the concentration that caused 50 % decrease in erythrocyte membrane lipid oxidation. IC_50_ values for BH and BS and for AA are provided in Table 5.

**Table 5:** IC_50_ values for two oxidation inducers (UVC and AAPH) in the presence of BH and BS extracts and for ascorbic acid (AA)

IC_50_ (mg ml^−1^)		
Compound	UVC	AAPH
BH	0.0300 ± 0.0027^a^	0.0100 ± 0.0005^c^
BS	0.0306 ± 0.0031^a^	0.0123 ± 0.0007^b^
AA	0.0166 ± 0.0026^b^	0.0201 ± 0.0011^da^

Different letters (a–c) within the same column indicate significant differences at *p* < 0.05 by Duncan’s test

^d^Data presented in our earlier publication: Cyboran et al. 2015

In the fluorimetric method, erythrocyte membrane lipid oxidation was induced chemically with the AAPH compound. As a result of AAPH break-up, free radicals developed that caused damage to the probe DPH-PA observed and decline in its fluorescence intensity. Antioxidant activity of the extracts was determined on the basis of their ability to protect the probe from damage, and thus to inhibit the oxidation of lipids in the MRBC. In Fig. 6c and d, the relative fluorescence of probe DPH-PA is shown. As can be seen from the figures, relative fluorescence decreases with time of oxidation and concentration, the decrease being slower as the extract concentration increases. To compare the antioxidant activity of the substances, like in the spectrophotometric method, the concentrations IC_50_ causing 50 % inhibition of membrane lipid oxidation were determined and together with those of AA are given in Table 5.

It has been shown that tested extracts protect the lipid MRBC when the oxidation was induced by UVC radiation as well as by the compound AAPH. In both methods, the antioxidant activity of BH and BS was close to each other with a small advantage for BH. For UV radiation, the activity of BH and BS was lower in comparison with AA, and for AAPH compound the activity of BH and BS was higher than AA (Table 5). No significant difference (*p* < 0.05) was found between antioxidant activity induced by UVC radiation for BH and BH extracts.

#### Anti-inflammatory Activity

Studies have shown that extracts of BH and BS, to varying degrees, inhibit the enzyme activity of cyclo-oxygenase 2 (COX-2). In these tests, as a measure of anti-inflammatory activity of tested substances were assumed concentration values (IC_50_) responsible for 50 % inhibition of COX-2 enzyme activity. The IC_50_ values obtained for the test substances are provided in Table 6. The results obtained were compared with indomethacin, which is the standard medicine with anti-inflammatory effect (Cyboran et al. 2015). As indicated by the test results (Table 6), a much greater anti-inflammatory activity shows BH compared with the activity of BS, because at a lower concentration it causes 50 % inhibition of COX-2.

**Table 6:** Values of IC_50_ concentrations for BS, BH, and indomethacin (IND) at which 50 % inhibition of cyclo-oxygenase activity occurs

Compound	IC_50_ (mg ml^−1^)
BS	0.3810 ± 0.0604^a^
BH	0.2010 ± 0.0432^b^
IND	0.0076 ± 0.0007^c^

Different letters (a–c) within the same column indicate significant differences as *p* < 0.05 by Duncan’s test

## Discussion

Buckwheat is known as a very good source of phenolic compounds (Fabjan et al. 2003; Kalinová et al. 2006; Kalinová and Dadáková 2006; Kalinová and Vrchotova 2009). Qualitative and quantitative analysis carried out showed the presence of polyphenolic compounds in the extracts from BS and BH (Table 1). 24 compounds have been identified in each extracts, including 22 flavonoids and 2 phenolic acids. The total content of polyphenolic compounds in BH extract amounted to 371.91 mg g^−1^, and in BH extract from approx. 1.5 times more, i.e., 547.13 mg g^−1^.

The hemolytic studies have shown that the extracts, in a wide range of concentrations, not only do not act destructively on the MRBC, without causing hemolysis of RBC, but strengthen it. The absence of hemolytic toxicity of different polyphenolic extracts is shown by Bonarska-Kujawa et al. (2014), Cyboran et al. (2012), and Suwalsky et al. (2007). These results allow believing that polyphenolic compounds present in the extracts do not penetrate deeply into the MRBC, since hemolytic action is caused by hydrophilic–hydrophobic molecules whose alkyl chains penetrate the hydrophobic region of the lipid bilayer, weakening the interaction between components of the membrane (Kleszczyńska et al. 1986; Łuczyński et al. 2013).

The results of osmotic resistance studies on erythrocytes treated with the extracts have shown that the erythrocyte membrane, possibly as a result of sealing, becomes more resistant to changes in osmotic pressure. A higher osmotic resistance of erythrocytes treated with other plant extracts is found by Cyboran et al. (2012, 2014), He et al. (2008b), and Wang et al. (2001). The increase in osmotic resistance of BS- and BH-modified RBC can be explained by the fact that polyphenolic compounds present in the extracts become incorporated mainly in the polar region of the lipid bilayer, as evidenced by the results of the fluorimetric research, shape, or FTIR studies presented in this paper, as well as our earlier work (Włoch et al. 2013). The presence of polyphenolic compounds in the outer part of the membrane reduces the penetration of water into the interior of the RBC driven by osmotic pressure difference.

Analysis of the shapes of red blood cells allows one to conclude that polyphenol compounds contained in buckwheat extracts incorporate mainly into the outer lipid monolayer. Echinocytes, developed as a result of extract–membrane interaction, testify to such location, according to the theory of lipid bilayer coupling (Deuticke 1968; Lim et al. 2002; Sheetz and Singer 1974). Bors et al. (2012) showed that a change in the shape and size of red blood cells depends on the total content of phenolic compounds in the extract. The results we got also showed that a greater content of polyphenolic compounds in the BS extract results in creation of more echinocytes compared with BH extracts (Table 2).

In experiments performed with MC540 probe, it was shown that MC540 binding is very sensitive to lipid packing of phospholipids bilayers. The research results showed that fluorescence of MC540 increases in the presence of loosely packed membrane compared to that in the presence of lipids in the gel phase (Alay et al. 2003; Manrique-Moreno et al. 2014). In our study, the enhanced fluorescence intensity of MC540 was observed for BS (Fig. 3). That increased intensity of MC540 suggests a decreased organization of lipids, which would indicate increased membrane surface area accessible for binding of the dye due to the loss of lipid packing (Langner and Hui 1999; Manrique-Moreno et al. 2014).

Fluorimetric study of erythrocyte membrane in the presence of buckwheat extracts showed a clear decline in the value of the GP, higher for the BS extract, indicating a significant increase in the disorder of membrane lipids in the hydrophilic region. Investigation of the hydrophobic area using the DPH probe showed a slight increase in fluorescence anisotropy (*A*) for the extract BH but no change for BS (Table 3). The BH extract caused a slight stiffening of the membrane in that area, which may be a consequence of modifications observed in the hydrophilic region. Higher concentrations of quercetin in BH extract may be responsible for the slight stiffening of the membrane. This may result from quercetin joining of the polar heads of phospholipids by hydrogen bonds and thus also affecting the alkyl chains (which may result in increased order of alkyl chains) (Pawlikowska-Pawlęga et al. 2014). Our previous studies, with respect to the unit lipid membrane model created of DMPC, showed that BH and BS extracts cause changes in both the outer area of the hydrophilic region and fluidity of the lipids in the hydrophobic region (Pruchnik et al. 2015).

In order to get confirmation of results obtained by fluorimetric method with fluorescent probes (Merocyanin, Laurdan and DPH), we conducted studies using the FTIR method.

The results obtained by FTIR confirm our observations on the location of the compounds in the membrane. An increase in hydration of the phosphate and carbonyl group suggests changes in the hydrophilic area. The results of the measurement of the coefficient of dichroism and the order parameter indicate an increase in disorder in the polar heads in the presence of the extracts, while in the chain area there is an increase in order. The angle between polar head and the normal to the membrane plane decreases in relation to the control. This means that the heads change orientation more vertically. In both cases, larger changes were observed in the presence of BH extract. Tested extracts may also have effect on membrane proteins, which is suggested by the changes we observed in the setting and order parameter of proteins observed by the dichroism method. On the basis of the changes observed in the degree of hydration and dichroism induced by the extracts, we can draw the conclusion that the ingredients contained in BH incorporate deeper into the membrane than the ones present in BS.

Biological activity of the extracts was determined on the basis of their anti-inflammatory and antioxidant activity.

Studies of antioxidant activity of the extracts showed that they protect the erythrocyte membrane against oxidation regardless of the kind of inducer (UVC radiation or AAPH compound). In both cases, the antioxidant activity of BH and BS was on a similar level, but the study has shown that BH and BS extract are much better antioxidants than AA in relation to the free radicals induced by AAPH compound (Table 5). Antioxidant activity of extracts from different parts of buckwheat were also studied by Gulpinar et al. (2012) and Sun and Ho (2005) using different inducers of oxidation and other research methods. There are virtually no reports concerning the antioxidant activity of extracts from BH and BS in relation to biological membranes and methods used. Only Mukoda et al. (2001) showed a good antioxidant activity of BH extract in erythrocyte membranes of the rat. Taking into account the results of the fluorimetric, FTIR, shape of erythrocytes, and antioxidant activity research, one can specify the likely molecular mechanism responsible for the antioxidant properties of the extracts. Polyphenolic compounds contained in the extracts as scavengers of free radicals reduce the amount of free radicals in the solution near the membrane, thus reducing the likelihood of their diffusion into the interior of the membrane. Therefore, we can say that the extracts constitute a kind of barrier that protects the membranes from oxidation by free radicals induced both by physical factors like UVC radiation and chemical ones like the AAPH compound.

In order to determine the anti-inflammatory activity of the extracts, we tested their effect on the inhibition of the enzyme COX-2. The studies have shown that both extracts reduce the activity of the enzyme, but a higher inhibition shows the BH extract (Table 6). This activity, however, is lower than that of indomethacin, a commonly used synthetic medicine. It can be assumed that the higher anti-inflammatory activity of BH is due to higher content of routine and quercetin in the extract, as these compounds significantly inhibit the expression of COX-2, suggesting its high anti-inflammatory activity (Kim et al. 2013; Liu et al. 2008).

## Conclusions

Extracts of husk and stalk of buckwheat are a rich source of polyphenolic compounds, the extract BS having more of these compounds. The hemolytic test results and those of osmotic resistance indicate that the extracts do not act destructively on the membrane but reinforce it, causing an increase in osmotic resistance of erythrocytes. The study of biophysical parameters of erythrocyte membrane have shown that polyphenols contained in the extracts become incorporated mainly in the hydrophilic part of erythrocyte membrane, constituting a kind of barrier for free radicals attacking the membrane from the solution. The presence of polyphenols in this part of membrane induces changes in the properties of the area, which may find reflection in small changes of fluidity of that area that we observed for BH extract. The biological activity of extracts from buckwheat specified in this study depends not only on the quantities of polyphenolic compounds but also on their kind.

The present research on the interaction of polyphenolic compounds with the erythrocyte membrane is important from the point of view of future uses of the compounds in medicine.

## Electronic supplementary material

Below is the link to the electronic supplementary material.

Supplementary material 1 (DOCX 13 kb)

Supplementary material 2 (TIFF 187 kb)

Supplementary material 3 (TIFF 118 kb)

Supplementary material 4 (TIFF 99 kb)

Supplementary material 5 (TIFF 166 kb)

Supplementary material 6 (TIFF 159 kb)

## Abbreviations

AA: L (+) ascorbic acid
AAPH: 2,2′-azobis(2-amidinopropane)hydrochloride
BH: Buckwheat husk extract
BS: Buckwheat stalk extract
COX: Cyclo-oxygenase
DPH: 1,6-diphenyl-1,3,5-hexatriene
DPH-PA: 3-(4-(6-phenyl)-1,3,5-hexatrienyl) phenylpropionic acid
Laurdan: (6-dodecanoyl-2-dimethylaminonaphthalene)
MDA: Malondialdehyde
MC540: Merocyanine 540
MRBC: Red blood cell membrane
RBC: Red blood cell
TBA: 2-thiobarbituric acid
TCA: Tricholoroacetic acid
TMPD: Tetramethyl-*p*-phenylenediamine
