# Supplementary material for: Physical Effects of Buckwheat Extract on Biological Membrane In Vitro and Its Protective Properties
Source: J Membr Biol. 2015 Nov 18;249:155–70. doi: 10.1007/s00232-015-9857-y (PMC4851706; doi:10.1007/s00232-015-9857-y)
Supplement: Supplementary file 1 — Supplementary material 1 (DOCX 13 kb) [file 232_2015_9857_MOESM1_ESM.docx]

**List of supplementary material:**

Supplementary 1. Base peak chromatogram of buckwheat husk extract (refer to Table 1 for the identification of time retention peaks).

Supplementary 2. UPLC-MS chromatogram of catechin derivative in buckwheat husk extract (refer to Table 1 for the identification of each number peak).

Supplementary 3. UPLC-MS chromatogram of 1-O-caffeoyl-6-O-alpha-rhamnopyranosyl-beta-glycopyranoside in buckwheat husk extract (refer to Table 1 for the identification of number peak).

Supplementary 4. UPLC-MS chromatogram of gallate derivative of proanthocyanidin in buckwheat husk extract (refer to Table 1 for the identification of each number peak).

Supplementary 5. UPLC-MS chromatogram of flavonol and flavan derivatives in buckwheat husk extract (Refer to Table 1 for the identification of each number peak).
