# Supplementary figures and images for: Physical Effects of Buckwheat Extract on Biological Membrane In Vitro and Its Protective Properties
Source: J Membr Biol. 2015 Nov 18;249:155–70. doi: 10.1007/s00232-015-9857-y (PMC4851706; doi:10.1007/s00232-015-9857-y)

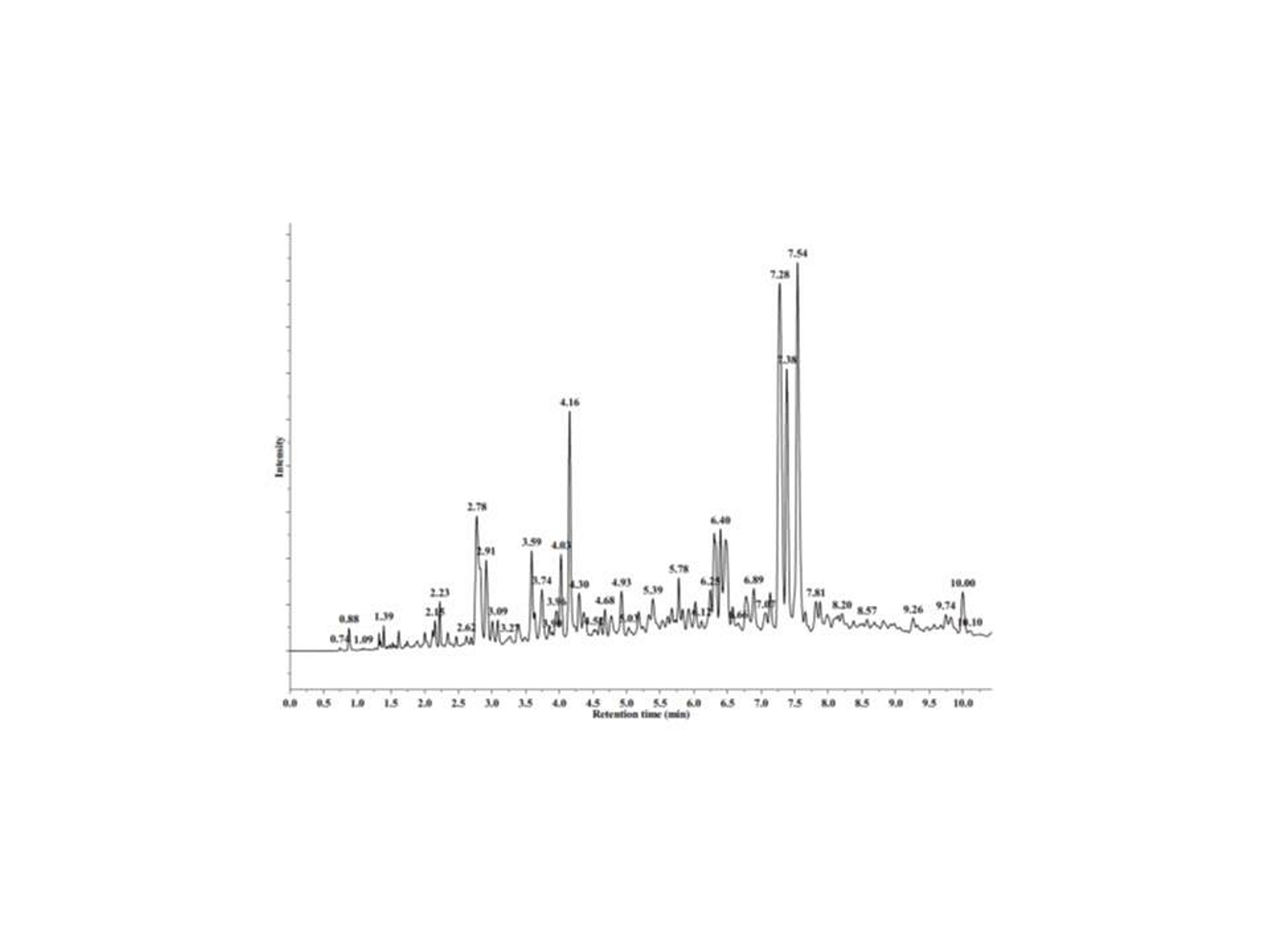

Supplement: Supplementary file 2 — Supplementary material 2 (TIFF 187 kb) [file 232_2015_9857_MOESM2_ESM.tif]

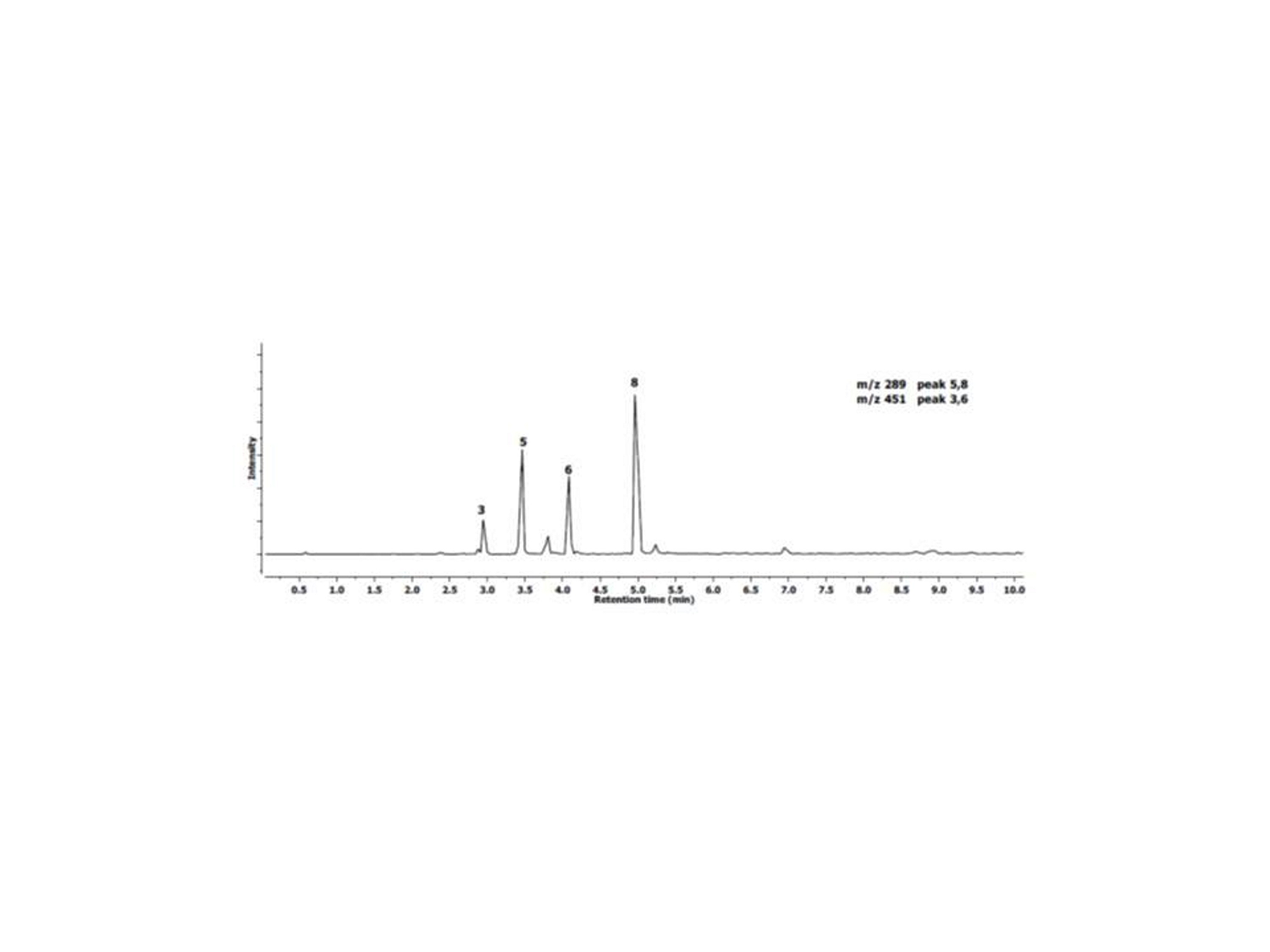

Supplement: Supplementary file 3 — Supplementary material 3 (TIFF 118 kb) [file 232_2015_9857_MOESM3_ESM.tif]

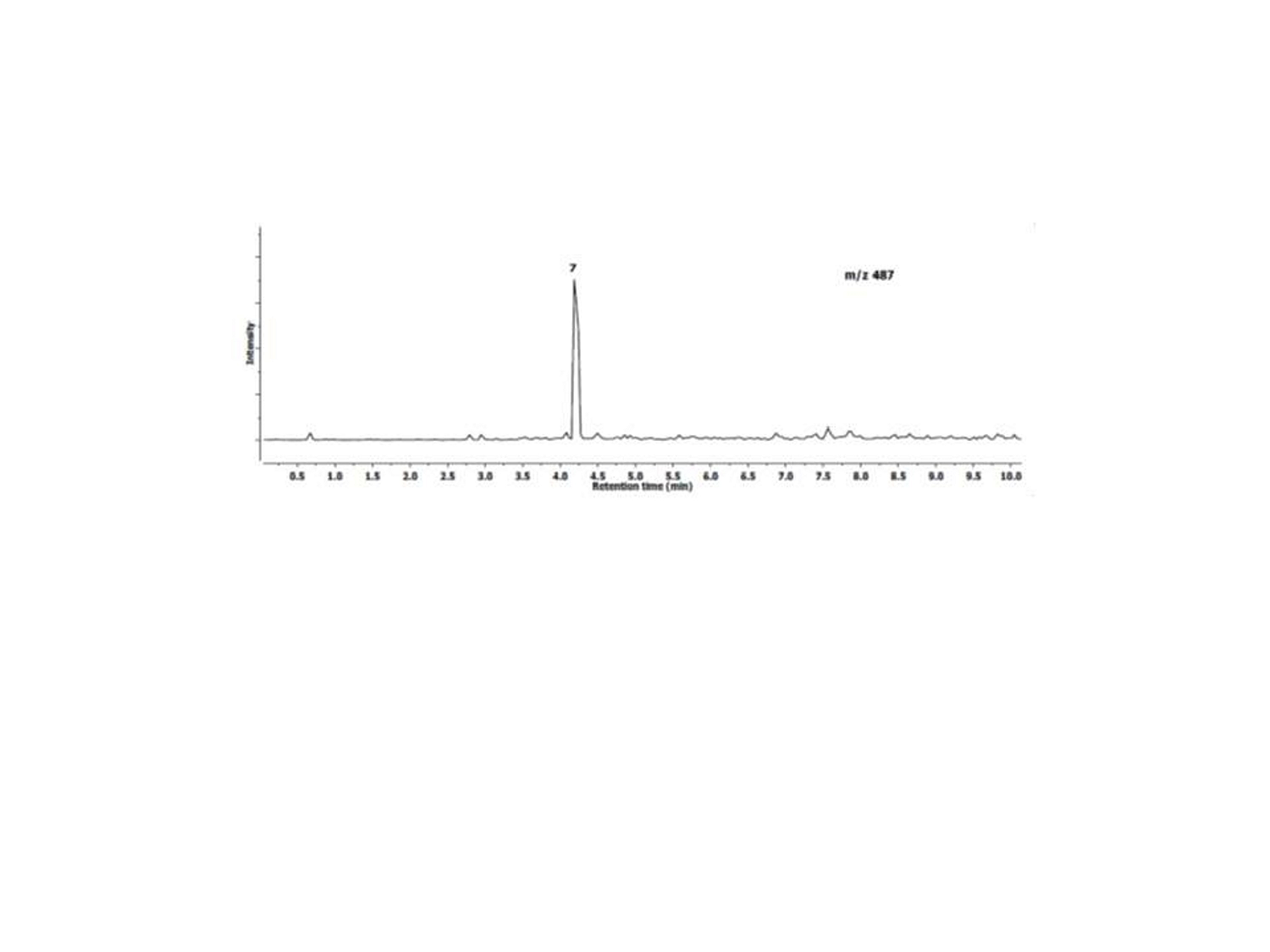

Supplement: Supplementary file 4 — Supplementary material 4 (TIFF 99 kb) [file 232_2015_9857_MOESM4_ESM.tif]

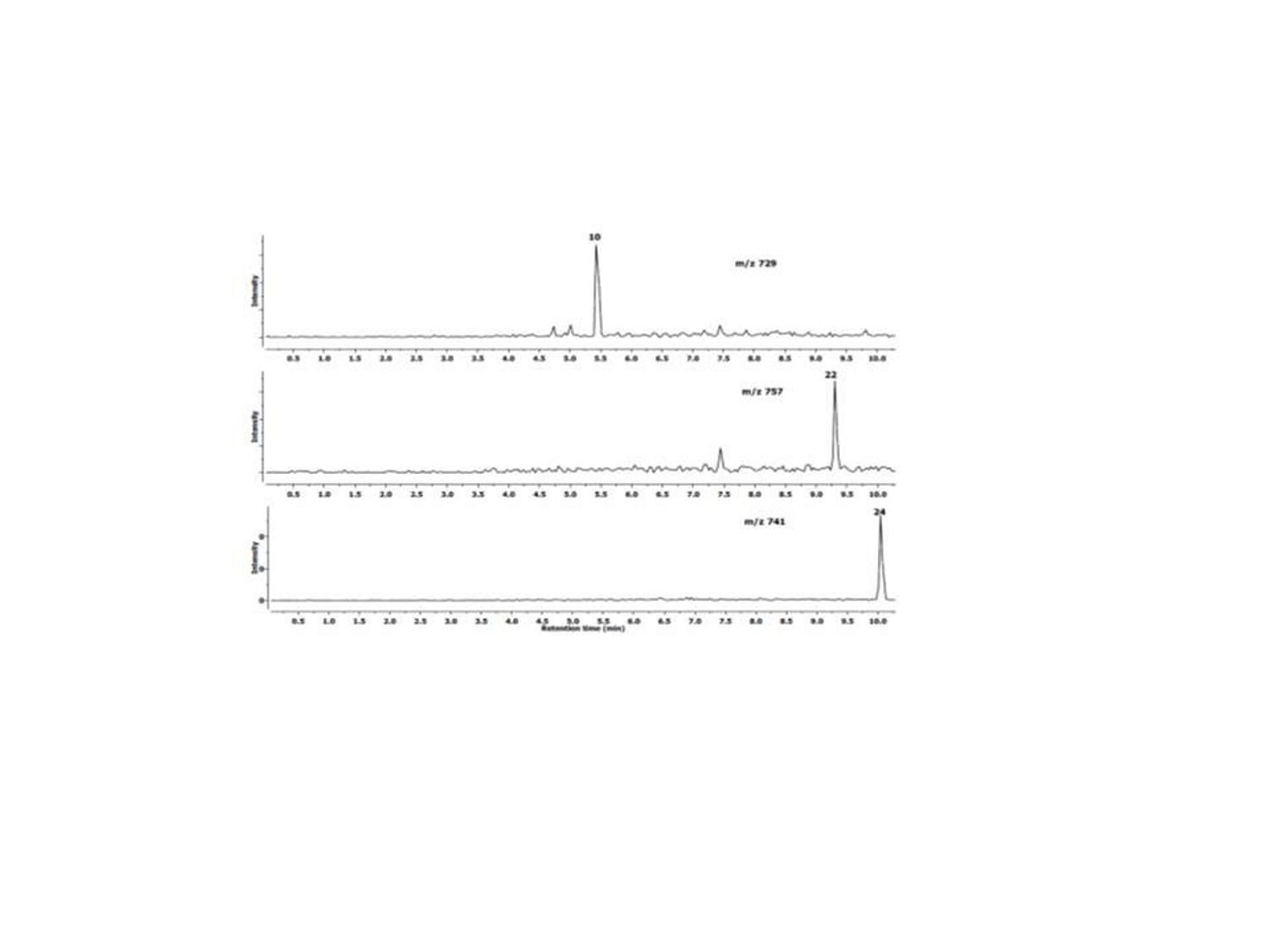

Supplement: Supplementary file 5 — Supplementary material 5 (TIFF 166 kb) [file 232_2015_9857_MOESM5_ESM.tif]

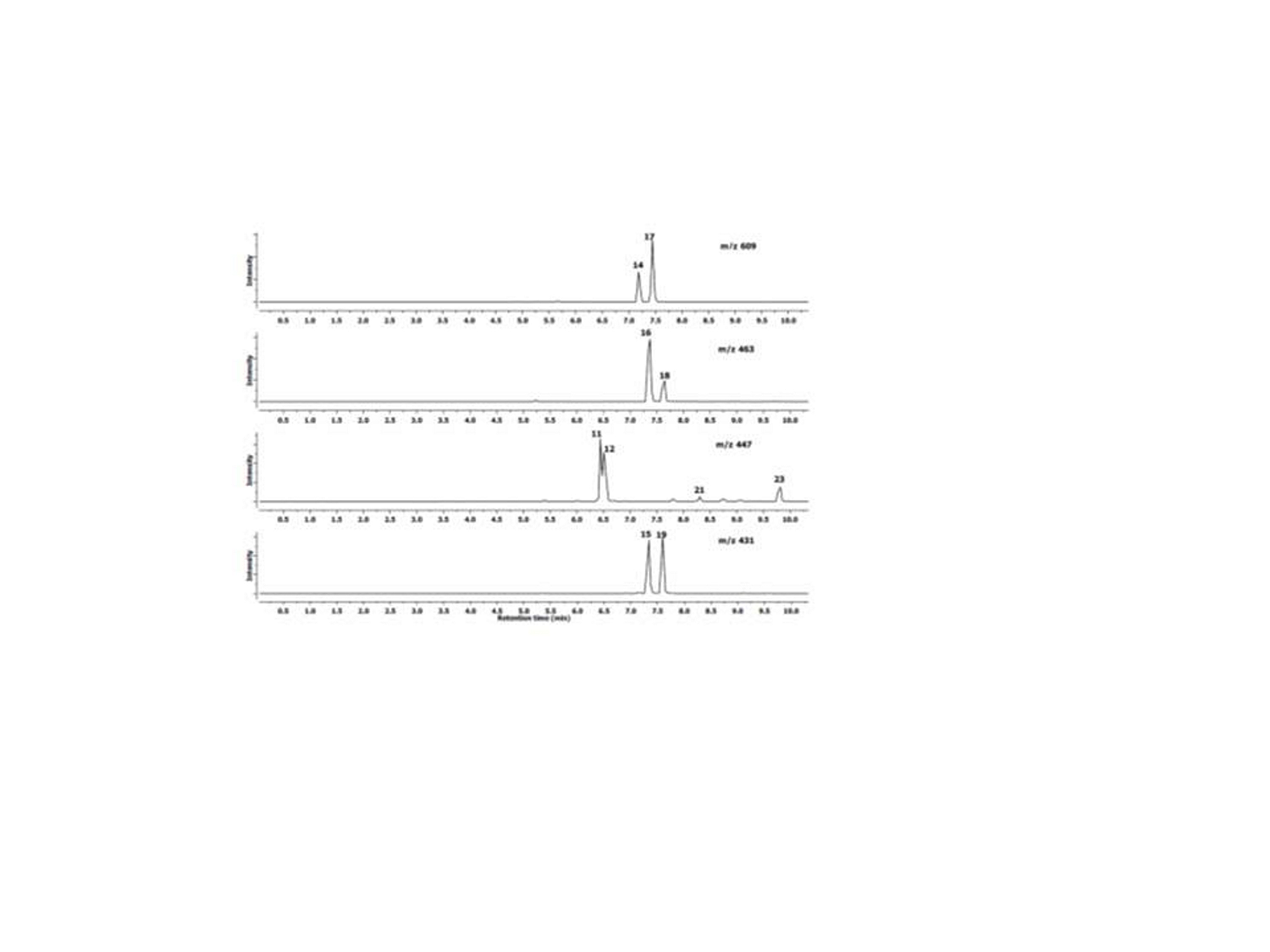

Supplement: Supplementary file 6 — Supplementary material 6 (TIFF 159 kb) [file 232_2015_9857_MOESM6_ESM.tif]
